# Rules, Norms and Practices – A Comparative Study Exploring Disposal Practices and Facilities in Northern Europe

**DOI:** 10.1177/00302228211042138

**Published:** 2021-09-08

**Authors:** Helena Nordh, Danielle House, Mariske Westendorp, Avril Maddrell, Carola Wingren, Sonja Kmec, Katie McClymont, Christoph Jedan, Tanu Priya Uteng, Yasminah Beebeejaun, Eric Venbrux

**Affiliations:** 1 Department of Urban and Rural Development, Swedish University of Agricultural Sciences, Uppsala, Sweden; 2 Department of Geography and Environmental Sciences, University of Reading, Reading, United Kingdom; 3 Department of Religious Studies, University of Groningen, The Netherlands; 4 Department of Humanities, University of Luxembourg, Belval, Luxembourg; 5 Geography and Environmental Management, University of the West of England Bristol, Bristol, United Kingdom; 6 Department of Mobility, Institute of Transport Economics, Oslo, Norway; 7 The Bartlett School of Planning, University College London, London, United Kingdom; 8 Centre for Thanatology/Department of Comparative Religion, Radboud University, Nijmegen, The Netherlands

**Keywords:** burial, grave, cemeteries, crematoria, multicultural societies, religious diversity

## Abstract

We identify and analyse practices and management regimes around burial and handling of ashes across eight case study towns within six Northern European countries. We analyse management of cemeteries and crematoria gardens, majority practices and provision for minority communities, including various burial types, cremated remains, the re-use of graves, and costs for interments. Comparative data is drawn from analysis of national and local regulations, interviews with stakeholders, and observations at cemeteries and crematoria gardens. The findings show significant variation in national and local regulations and practices for burial and cremation particularly around the re-use of graves, handling of ashes and costs for grave space and cremation. We identify the opportunities and constraints of these variations in terms of accessibility, diversity and equality; and argue for national directions to avoid unequal treatment within nations. Furthermore, we stress the importance of a liberal and inclusive management of European cemeteries and crematoria gardens.

## Introduction

To set the scene for the following discussion we begin this paper with an anecdote from our fieldwork. In Luxembourg-city, one of our case studies, we interviewed an Irish migrant in her 50s, referred to here as ‘Mary’. During our conversation she mentioned how shocked she was that in Luxembourg-city bodily remains are removed from graves when the rental period expires. For Mary, this was continually surprising, and even after two decades of living in Luxembourg, she reported still being shocked about the disinterment and grave re-use *every time* she heard about it. Growing up in the Republic of Ireland, her experience of Irish funerary practices, including an expectation of permanent graves, shaped her ideas of what is ‘normal’ or ‘common’ practice. Her experience exemplifies Tony Walter’s (2005, p. 173) observation that:

… national practices are so taken for granted that members of one society are typically amazed and even appalled that neighbouring countries organize the disposal of their dead differently, yet so far there has been very little scholarly analysis of such differences.

In most Northern European contexts, people’s experiences of cemeteries or crematoria gardens are limited to attending funerals and memorial practices for kin or close friends, or maybe using them as local green spaces. Consequently, experiences are typically limited to specific localities and the attendant norms of practices within those contexts. In this paper, we identify and compare contemporary burial and ash disposal practices in six Northern European countries: Ireland, Scotland, The Netherlands, Luxembourg, Sweden, and Norway. In-depth studies were conducted in eight midsized towns across these countries (see Figure 9) chosen as examples of religious and cultural traditions in Northern Europe. In the analysis, we focus on the following topics related to the management of cemeteries and crematoria gardens: practice and provision for majority and minority communities (including various burial types and protocols for cremated remains), the duration of grave rights or re-use of graves, and the costs of burials and cremations. The paper will serve as a systematic comparison of management of cemeteries and crematoria gardens in Northern Europe. With numerous examples we illustrate how cemeteries and crematoria gardens are organised and managed vary markedly between countries, and even towns, regardless of their relatively similar cultural histories and contemporary social structures, such as welfare provision. This comparative study provides a foundation for discussing how different systems hinder or promote accessibility, diversity and equality in cemeteries and crematoria gardens. This allows cemetery research to move beyond the typical national focus, providing an increased understanding of similarities and differences across countries. In addition, throughout the article we highlight examples and ideas which can inform policy and practice at a local level.

### Some Notes on Terminology

We use the terms ‘cemeteries’ and ‘crematoria gardens’ to describe the various spaces for the disposal of dead bodies examined in our case studies. The terminology used around the disposal of bodily remains in Europe reflects a combination of religious, traditional, and cultural practices, public health concerns, and other factors (Burkette, 2015). The term ‘crematoria gardens’ is used to refer to the area surrounding a crematorium building where ashes are buried or dispersed, such as meadow areas, urn graves, or columbaria.

As multiple scholars have shown (e.g., Clayden et al., 2015; Rugg, 2000, 2020; Yarwood et al., 2015), the term ‘cemetery’ and what it means varies widely from one country to another, to include faith-based burial grounds, municipal post-secular cemeteries, woodland burial sites and graveyards, memorial gardens, and crematoria gardens.

Recently in Norway and Sweden the legal wording that describes a space for the disposal of remains in the national Burial Acts was changed from churchyard *(kirkegård* in Norwegian*, kyrkogård* in Swedish*)* to cemetery (*gravplass, begravningsplats*). In both countries, this was a step towards the separation between the Church and State and an attempt to address the needs of their postsecular societies which include a parallel growth of secularisation and a greater diversity of religious beliefs and practices (see more on Scandinavian cemetery management in the results section below). In line with this context, Scandinavian studies refer to both cemeteries and churchyards (cf. Kjøller, 2012; Nordh & Evensen, 2018). In the Dutch language, the term cemeteries (*begraafplaatsen*) similarly encompasses churchyards, municipal and private cemeteries, as well as woodland and natural burial sites. Similarly, in the Luxembourgish language, one word is used to describe all types of spaces for disposal of human remains (*Kierfecht* in Luxembourgisch or *cimetière* in French), which also includes forest cemeteries (Kmec & Kolnberger, 2020). In Scotland and Ireland, the term ‘churchyard’ tends to be limited to Christian burial grounds and for this study we have focused on municipal post-secular cemeteries as distinct from historic graveyards in church grounds; see Rugg (2000) on the specific historical conditions from which the modern cemetery emerged in the UK and Ireland.

In the next section, the broad points of convergence of the religious-cultural contexts of the case study countries are briefly outlined and some variances identified, followed by an analysis of the management of cemeteries and crematoria gardens in the Northern European context.

### The Northern European Context

#### (Post)Secular Societies

The countries in this study share the same broad religious-cultural heritage shaped by varied Christian traditions (notably Reformed and Catholic churches) and Enlightenment ideas of rationality (also see Kolnberger, 2018 on European traditions). They are also marked by post-war trends towards (post)secularization and increased religious diversity (Beaumont & Baker, 2011; Habermas, 2010; McLennan, 2010; Molendijk et al., 2010), which has likewise affected attitudes and mentalities towards death and funerary practices (Ariès, 1974; Jacobsen, 2016; Klass & Steffen, 2018; Maddrell, Beebeejaun, McClymont, McNally, et al., 2018; Mathijssen, 2017). The understandings of secularization and postsecularization vary across countries and scholars. Where secularization was often understood as a general decline in religion, postsecular scholars recognize that the public role of religion has instead changed (Kjærsgaard, 2017; Molendijk et al., 2010). In the countries under investigation, spirituality is very much present in people’s everyday practices (Beaumont & Baker, 2011; Berghuijs et al., 2013; Kjærsgaard, 2017). While traditional religious affiliations (most notably Christian) are declining, some people with limited affiliation to religious institutions continue to find comfort in traditional religious funerals. Others find their spiritual and religious needs met in other contexts and communities (McClymont, 2015), such as Buddhism, and self- or nature-based spiritualties and other alternative belief systems. It has been argued that these changes are reflected in an increased interest in cremation and varying alternatives for burying or scattering ashes (Harvey, 2016; Heessels et al., 2012; Van der Velde, 2013; Walter, 2020), but this can present an over-simplified elision of individualisation and alternative disposition with secularisation (Maddrell, 2011). Both secular and increasingly diverse religious beliefs and practices characterise post-secular societies’ funerary ideals and practices, including the blending of secular and religious practices, or those from different religious traditions (Maddrell, Beebeejaun, McClymont, McNally, et al., 2018).

#### Management of Burial and Cremation

In each case study country, the proper management of the dead is understood to be of national significance, and national legal frameworks are set up to provide the basis for the management of the deceased.^1^ However, despite these legal frameworks, local differences abound. Numerous scholars have attempted to categorise funerary practice in the West. Drawing on social justice theory, Julie Rugg (2020) argues burial is a *necessary social service*. She describes how cemetery systems differ between countries, but in many European countries have shifted from the responsibility of the Church or other religious organisations to the state. In order for cemeteries to be socially just, they have to be able to provide a decent disposal of the body, democratic accountability, equality of access to services regardless of income, freedom of religious expression at cemeteries, and environmental sustainability (Rugg, 2020). An additional element can be identified, namely specific provisions for religious and minority communities (Hunter, 2016; Maddrell, Beebeejaun, McClymont, Mathijssen, et al., 2018, Maddrell, Beebeejaun, McClymont, McNally, et al., 2018; Wingren, 2013). Attention to diverse religious and cultural funerary practices has been shown to be central to culturally inclusive cemeteries and crematoria gardens as well as the sense of ‘full citizenship’ of minorities, but can also be a cause of contention (Maddrell, Beebeejaun, McClymont, Mathijssen, et al., 2018; Maddrell, Beebeejaun, McClymont, McNally, et al., 2018; Maddrell, McNally, et al., 2021). Tony Walter (2005), focusing on Western Europe and North America, describes three models of *commercial*, *municipal*, and *religious* funeral organisation, relating to “the management of the corpse until its final disposition” (Walter, 2005, p. 173). The commercial model refers to cemeteries managed by private businesses. The municipal model suggests they are managed and owned by the municipality or other state-run organizations, and in the religious model, religious organisations are responsible for funerals, cemeteries, and crematoria. Walter likewise acknowledges that mixed-models emerge that combine these three. We draw on this preceding work in the analysis of the modes of management of cemeteries and crematoria gardens in the selected case study towns.

#### The Role of Cemeteries and Crematoria Gardens

**Figures 1–3. Examples of cemeteries in Scandinavia.**
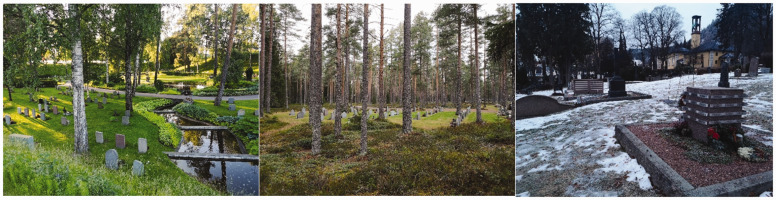
From the left to right: photos from the main cemetery St Eskil in Eskilstuna; woodland cemetery Röbäck in Umeå; collective memorial at the central cemetery Bragenes in Drammen, the yellow building in the back is the crematorium (photographs by Helena Nordh).

Cemeteries and crematoria gardens across Northern Europe are generally designed and managed as ‘green spaces’: that is to say they are landscapes (gardens and parks) shaped by local contexts such as cultural heritage, recreational needs, and secularism (Wingren, 2013), and are designed by a landscape architect and/or developed over time through pragmatic management processes. Nonetheless, there are key differences between cemeteries. Historically, cemeteries in Protestant countries are typically marked by trees, shrubs, flowers and grass, whereas cemeteries in Catholic nations generally rely on hard surfaces (Walter, 2020). Furthermore, woodland and natural burial grounds are more prominent in historically Protestant countries than in Catholic ones (Walter, 2020). However, the picture is more nuanced and depends in part upon intersecting cultural norms, funding streams and other practicalities, rather than simply reflecting denominational dichotomies. Many western historic cemeteries have mature trees (Quinton et al., 2020) and function as valuable open spaces (Curl, 1975). They are commonly categorised by planners as green infrastructure in cities (see McClymont, 2016 on the UK and Nordh & Evensen, 2018 on Scandinavia), and are managed as such (Kjøller, 2012). Some cemeteries are even used as public parks (Evensen et al., 2017; Grabalov, 2018; Maddrell, Beebeejaun, McClymont, Mathijssen, et al., 2018; Maddrell, Beebeejaun, McClymont, McNally, et al., 2018; Skår et al., 2018). In Figures 1 [Fig fig2-00302228211042138]to 8 we have selected typical photos of cemeteries from each of the case study towns. As evident in these pictures, all have elements of nature, regardless of their religious heritage. However, the design of the spaces and the amount of natural elements, is dependent on other aspects such as topography and local and national regulations, and therefore varies across countries and cemeteries. For more on design of cemeteries in the resective countries see for example Dietze-Schirdewahn and Lunde (2019), Nolin (2006), Wingren (2013) on Scandinavia; see Kmec et al. (2019) on Luxembourg; Tarlow, 2000 on the UK; Deunk et al. (2016); Van Raak (1995) on the Netherlands.

**Figures 4–5. Examples of cemeteries in Ireland and Scotland. fig2-00302228211042138:**
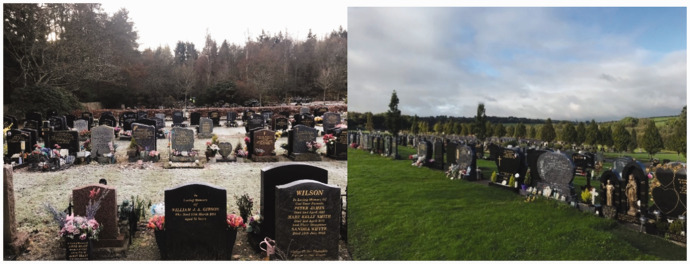
From left to right: photos from Birkhill cemetery in Dundee; St James’ cemetery in Cork (photographs byDanielle House).

**Figures 6–8.**
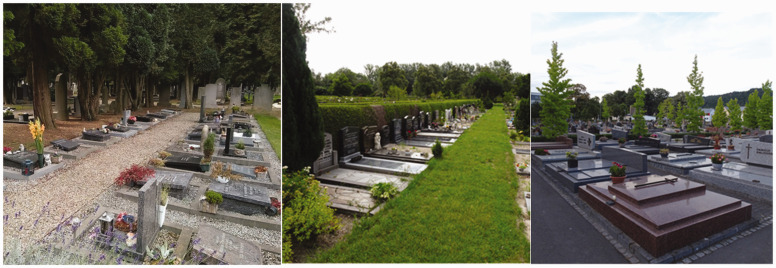
**Examples of cemeteries in The Netherlands and Luxembourg.** From left to right: photos from Tongerseweg municipal cemetery in Maastricht; municipal cemetery Noorderbegraafplaats in Leeuwarden; Notre Dame in Luxembourg-City (photographs by Mariske Westendorp).

Across our case studies cemeteries and crematoria gardens can be regarded as public spaces where everyday encounters are made (Francis et al., 2000; Grabalov & Nordh, 2021; Maddrell, Beebeejaun, et al., 2021; Maddrell, Beebeejaun, McClymont, Mathijssen, et al., 2018; Maddrell, Beebeejaun, McClymont, McNally, et al., 2018; Maddrell, McNally, et al., 2021; Swenson & Skår, 2018), which can result in conflict between different interests and practices, or community for example through spaces that gather people with joint needs or interests. The cemetery has been described as a liminal space in numerous ways, including as a “floating border between private-public spaces” (Swensen & Brendalsmo, 2018, p. 88): even if most are publicly accessible spaces, they provide ‘private’ burial plots that are bought or rented and are commonly treated more or less as “miniature home gardens” (Kjærsgaard & Venbrux, 2016) where “memory objects … form a specific passage landscape between life and death” (Maddrell et al., 2021, p. 8). Moreover, what is permitted within these spaces in terms of behaviour, memorial, and burial practices is regulated nationally and/or locally and not least as normative “unwritten rules” in people’s minds (Nordh et al., 2017).

As places of mourning, bereavement, and consolation, cemeteries and crematoria gardens accommodate personal as well as social and environmental functions (Jedan et al., 2019). The meaning of the individual/private grave, and how it is shaped and decorated, is of importance for mourning and remembrance (Petersson & Wingren, 2011). Further, interactions with ‘deathscapes’ are not just experienced through the materiality of physical spaces, but also and at the same time through embodied-psychological experience and various forms of virtual space, including both digital spaces and arenas of belief and belonging, such as idea of ‘heaven’ (Maddrell, 2016). This embodied experience of loss, mourning, and consolation is also frequently reflected in a sense of a continuing bond with, and/or responsibility to, the dead (Klass et al., 1996; Klass & Steffen, 2018). This highlights the complexity of emotional-affective environments, particularly those connected to the disposition of the dead, mourning, and remembrance, and how this is culturally inflected. This in turn demonstrates the social and cultural significance of cemeteries and crematoria gardens, and the importance of their organisation and management, including their diversity-readiness .

## Methods

**Figure 9.**
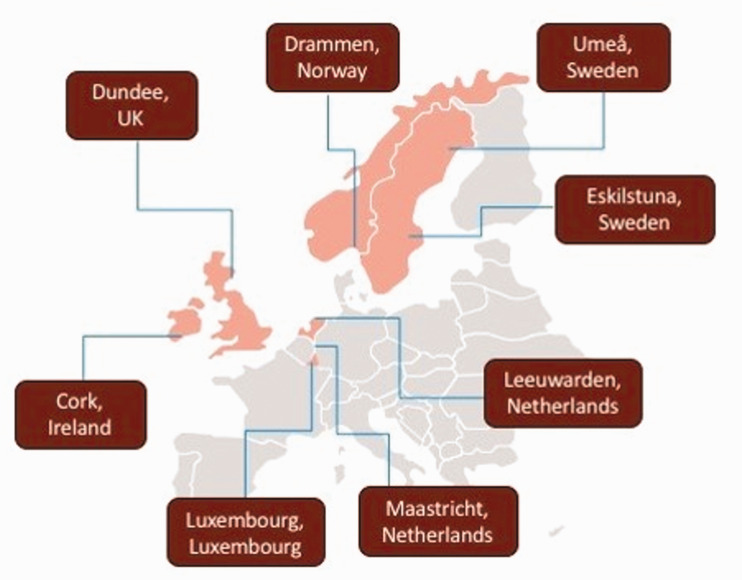
An Overview of the Eight Case Study Towns in Six Countries.

This paper is based on a comparison of six northern European countries and exemplified with in-depth studies in eight medium sized municipal areas or ‘towns’ (see Figure 9). This focus on medium-sized urban areas is designed to extend knowledge of provision for minorities and migrants beyond large multicultural conurbations. The case studies were chosen for their medium-size, from a national perspective, with a significant proportion of minority or migrant populations (see Table 1). Their size also meant it was possible to survey all cemetery and crematoria garden provision, rather than sampling, affording an overview of total provision in the towns. Only cemeteries and crematoria gardens that are currently in active use for disposition are included in the study.

**Table 1.** Case Study Overviews.

Case study town (nation)	Population in the town or municipality/% ‘minority’ population (year)^a^	Top 5 key minority groups within the town or municipality^a^	Main cemetery and crematoria providers in/near town (religious, municipal, commercial)	Cemetery minority provisions	Ash scattering provision
Cork (Ireland)	125,657/14% (2016). A boundary change in 2019 increased the population to 210,000.	Polish: 2.6% British/Northern Irish: 1.5% Lithuanian: 0.4% Other EU: 5.3% Asian: 2.8%	Since the 2019 boundary change, 13 cemeteries: all municipal. Additionally, countless religious churchyards (Roman Catholic and Church of Ireland).One crematorium in Cork County (rural municipality adjacent to the city) that services Cork city: commercial.	Muslim burial in St Mary’s Passage West, Cork County Council, and St James’ Chetwynd, Cork City Council. Jewish burial Curraghkippane municipal cemetery (Cork City Council), section privately owned.	In Cork there is no provision for crematoria gardens or ash-scattering sections. However, cremated remains can be buried or scattered at the municipal cemeteries. There are no restrictions in regards to storing or scattering cremated remains. There is one columbarium in Cork and plans for new ones.
Drammen (Norway)	68,945/22%(2019)	Polish: 2.7% Turkish: 1.7% Iraqi: 1.2% Afghan: 1.1% Pakistani: 0.9%	Seven cemeteries: all managed by the Church of Norway. One crematorium: run by the Church of Norway.	Muslim burial (Skoger cemetery)	In Drammen, ashes have to be buried at the cemetery. However, there is possibility to apply for ashes to be spread in nature or over sea.
Dundee (Scotland)	148,750/10%(2018)	White Other: 4.7% Mixed or Multiple Ethnic Groups: 0.5% Asian, Asian Scottish or Asian British: 4.0% African: 0.8%	Six cemeteries: all municipal; One religious/private Muslim cemetery. One natural burial ground outside the city and one natural burial section within a municipal graveyard.One crematorium with a crematorium garden: commercial.	Muslim burial (Eastern and Birkhill municipal cemeteries and a private Muslim cemetery), Jewish burial (Eastern cemetery), Chinese section (Pitkerro Grove).	In Dundee, there are various sections within the crematorium garden for the burial or scattering of ashes, and it is possible to scatter or bury cremated remains in grave plots at municipal cemeteries. Scotland also allows private arrangements for storage or dispersal.
Eskilstuna (Sweden)	105,924/26% (2019)	Iraqi: 5.4% Finnish: 4.0% Syrian: 3.6% Somalian: 1.9% Eritrean: 0.8%	Two cemeteries: both managed by the Church of Sweden. One crematorium: operated by the Church of Sweden.	Muslim Section, Bahá'í Section, Mandei (St Eskil cemetery).	In Eskilstuna, ashes have to be buried at the cemetery. However there is possibility to apply for ashes to be spread in nature or over sea. There is also a local discussion to open up spreading of ashes at St Eskil cemetery.
Leeuwarden (Netherlands)	124,056/17% (2019)	Surinamese: 7.3% Antillean: 7.0% Moroccan: 5.3% Turkish: 2.6%	Eight cemeteries: 7 municipal, 1 religious (Roman Catholic); Two crematoria: both commercial. One of these has a crematoria garden.	Muslim section at Noorder Cemetery, Jewish section at Spanjaardslaan cemetery	In Leeuwarden, ashes can be scattered or placed in columbaria and grave urns at crematoria gardens and cemeteries, or can be taken home, spread in nature, used in material objects, repatriated etc.
Luxembourg-city, (Luxembourg)	122,273 /71%^b^ (2019)	French: 17.0% Portuguese: 11.0% Italian: 6.9% Belgian: 4.1% German: 3.7%	13 cemeteries: all municipal, including one forest cemetery.One crematorium with a crematorium garden: intermunicipal.	Muslim and Jewish sections(Merl cemetery).	In Luxembourg-city, ashes need to be scattered at the crematorium or local cemeteries, or placed in urn graves or columbaria at cemeteries. Provisions for ash scattering in the sea are also allowed.
Maastricht (Netherlands)	120,354/11% (2020)	Moroccans: 4.9% Turkish: 4.1% Antilleans: 1.7% Surinamese: 1.0%	11 cemeteries: 10 religious (Roman Catholic), one municipal; One crematorium: commercial	Muslim, Jewish and Armenian sections (Tongerseweg cemetery); Moluccan section (Bovens cemetery).	In Maastricht, ashes can be scattered or placed in columbaria and grave urns at crematoria gardens and cemeteries, or can be taken home and used for other purposes just as in Leeuwarden.
Umeå (Sweden)	127,119 /12% (2019)	Finnish: 1.7% Iraqi: 0.7% Iranian: 0.6% Somalian: 0.5% German: 0.4%	Four town cemeteries: all managed by the Swedish Church.One crematorium: operated by the Swedish Church.	Bahá'í section and Catholic section and planned Muslim section (Röbäck cemetery); Muslim section (Norra cemetery).	In Umeå, ashes have to be buried at the cemetery. However, there is possibility to apply for ashes to be spread in nature or over sea.

*Note*. Columns two and three are based on census data from the countries, which is why minorities are identified by different categories across the countries.

a The presented censuses are retrieved as follows: Cork 2011: https://corkhealthycities.com/wp-content/uploads/2019/01/CC-Profile-Report-Section-1-Part-I.pdf. Dundee 2016: https://www.dundeecity.gov.uk/sites/default/files/publications/Dundee%20Census%20Pofile%202011.pdf. Drammen 2019: https://www.ssb.no/statbank/table/09817/. Eskilstuna and Umeå 2019: https://www.scb.se/hitta-statistik/statistik-efter-amne/befolkning/befolkningens-sammansattning/befolkningsstatistik/pong/tabell-och-diagram/helarsstatistik–kommun-lan-och-riket/utrikes-fodda-efter-lan-kommun-och-fodelseland-31-december/. Maastricht 2020: https://maastricht.incijfers.nl/dashboard/bevolking-3/. Luxembourg 2019: https://www.vdl.lu/en/city/a-glance/facts-and-figuresLeeuwarden 2019: https://allecijfers.nl/gemeente/leeuwarden/

b The relative high number of migrants in Luxembourg can be explained by bilateral agreements with Portugal, Spain and Yugoslavia from the 1970s onwards; the nation-wide booming economy since the 1980s, attracting qualified workers from abroad in particular the financial and IT sectors; and Luxembourg-city's status as one of three EU capitals, making it home to many people working for EU institutions.

Mixed methods have been used, starting with an extensive in situ mapping of the cemeteries and crematoria gardens provision within each of the eight towns, including the layout and management of cemeteries or crematoria gardens, designated sections for religious and other minority communities, and the presence of columbaria and designated areas for scattering cremated remains. This systematic mapping and photo documentation allowed us to have a detailed understanding of the spatial and organisational similarities and differences between the cemeteries and crematoria gardens under study. 45 semi-structured in-depth interviews were held with stakeholders across the eight towns, including cemetery and crematoria managers and employees, town planners, and members of the municipal authorities.^2^ Most interviews lasted 60 to 90 minutes, were recorded, transcribed and coded. Shared open and thematic coding was undertaken using Atlas.ti. The data gathered from these interviews, observations, and systematic mapping was supplemented with analysis of national, local, and cemetery/crematoria gardens level regulations and local strategies or plans.

For the purposes of this paper, the following themes have been identified and will be discussed in detail in the following sections:

- Types and management of cemeteries and crematoria gardens (religious/municipal/commercial/other/mixed) (nationally/locally)
- Burial/cremation ratio (nationally/locally)
- Rules around tenure and the re-use of graves (nationally)
- Regulations for handling of cremated remains (nationally)
- Types of burial and arrangements for the scattering of cremated remains (nationally/locally)
- Costs for grave space (locally) including financial model of cemeteries
- Cemetery sections for minorities (locally)

## Findings: Exploring Similarities and Differences Across the Cases

In this section, we explore similarities, and differences across our case countries that came out of the comparative analysis. Tables 1 and 2 provide an overview of the different contexts and practices in the countries and towns.

**Table 2.** Overview of Cremation Ratio, Burial Provision, Length of Grave Rights and Costs for Grave Space Across the Countries in the Study.

Country	Cremation ratio nationally (year)	Burial provisions and ash scattering nationally	Length of grave rights nationally	Average grave costs in case towns	Average costs for cremation in case towns
Ireland	19.61% (2017)	Coffin graves (family graves most usual), urn graves and internment, columbaria (very rare).	Perpetual	1,800–2,000 € in Cork City Council municipal cemeteries. 1,000 € in Cork County Council municipal cemeteries.	In Cork there is one crematorium. The cost for cremation is 745 €.
Luxembourg	61% (2018)	Coffin graves (including family graves), urn graves, ash scattering, columbaria (relatively rare), natural burial.	15–30 years; perpetuity is only possible for religious reasons.	1,500 € for 30 years.	In Luxembourg, there is one crematorium. The cost for cremation is 1,375 €.
Netherlands	66% (2018)	Coffin graves, urn graves, ash scattering, columbaria, natural burial; other options with ash also possible (e.g., in jewelry or tattoos, or scattering in gardens).	No national regulations; dependent on individual cemeteries anywhere between 10-50 years; perpetuity is possible but expensive (around 10.000 €) and varies across cemeteries.	3,500 € for 20 years	Cost for cremation is decided locally. There is as of yet no crematorium in Maastricht. The crematoria in Leeuwarden have prices ranging from 1,375 to 1,550 €.
Norway	44% (2019)	Coffin graves, urn graves, scattering of ashes, from 1^st^ of January 2021 columbaria are introduced in the Norwegian burial law.	20 years, extension possible.	First 20 years covered by the home municipality, then a fee that varies across municipalities (in Drammen about 35 € per year).	In Drammen, the cost for cremation is covered by the municipality (via taxes).
Scotland/UK	77% (2017)	Coffin graves (mostly family graves), urn graves, scattering ashes (relatively rare), columbaria are scarce.	Perpetual	About 700 € for a family plot in Dundee City Council municipal cemeteries, plus a ‘perpetuities’ management fee of ∼630 €.	In Dundee, the cost for cremation is about 1,235 €.
Sweden	82% (2018)	Coffin graves, urn graves, spreading of ashes (relatively rare), a few active columbaria.	25 years; extension possible.	First 25 years covered by the state via taxes, then a fee for about 100 € for another 15 years in both Eskilstuna and Umeå.	In both Eskilstuna and Umeå, as well as in the rest of the country, the cost for cremation is covered by the burial fee (individualised tax).

### Management of Cemeteries and Crematoria Gardens

Across the countries in the study, cemeteries and crematoria gardens are owned and managed in various ways (see Table 1). In this paper, we apply the previously presented models of *commercial*, *municipal*, and *religious* funeral organisation as introduced by Walter (2005) in order to analyse and reflect on provision in international contexts. In Scotland, the Netherlands, Ireland, and Luxembourg, the majority of cemeteries are owned and managed by municipalities. Alongside these, there are some religious cemeteries, owned and managed by majority and minority religious organisations, primarily Christian, Islamic, and Jewish. Lastly, there are some commercial cemeteries, including recently developed woodland or natural burial grounds, and crematoria gardens. Crematoria gardens in these countries (Scotland, the Netherlands, Ireland, and Luxembourg) tend to be either commercial or municipal.

In our study, the ownership and management of cemeteries in Norway and Sweden are notable as cemeteries and crematoria here are in the main owned and managed by the Lutheran Christian Churches on behalf of the state. In Sweden, taking care of bodily remains is a public service operated by the Swedish Church. However, employees working at the cemetery do not have to be church members, and burial in Scandinavian cemeteries is open to all regardless of faith. Furthermore, in Norway and Sweden, crematoria gardens do not exist as separate entities, as crematoria are located within cemetery grounds, and are therefore also run by the Churches. However, in Sweden the County Administrative Board (*Länsstyrelsen*) is closely involved in decisions regarding the development and management of cemeteries. Cemetery management in Norway and Sweden therefore blurs the line between religious and municipal management (cf. Walter, 2005).

Lines can also be blurred between religious and commercial cemeteries. In Maastricht (Netherlands), the manager of a Roman Catholic cemetery consistently referred to it as a ‘churchyard’, as it is owned and managed by the parish of a nearby Catholic church. However, being aware of the declining numbers of Catholics in the city as well as a growing preference for cremation over traditional burial, the manager was adamant that the cemetery was open to everybody seeking a place to bury a loved one. Consequently, the cemetery has Catholic, Protestant, and secular graves. Another example of this blurring of categories was found in Ireland, where municipal cemeteries are theoretically secular but discursively are often seen as Catholic, based on local norms, practices and the history of the country. Some municipal cemeteries in Cork were described by their managers as ‘Catholic’, others as having Catholic and Protestant sections, and others as non-denominational. One recently built cemetery was described by the manager as “non-denominational […] except for the Muslim section” and the manager of another recently opened cemetery referred to the main burial section as “the Catholic section,” in distinction from an adjacent private Jewish cemetery.

The financing of a cemetery or crematoria garden is determined by the management model, which is a topic that we will now briefly look into. In Scotland, Ireland and Luxembourg, municipal cemeteries are managed by the municipality and funded through fees for grave plots, whereas commercial cemeteries and crematoria gardens in Scotland and Ireland are financed purely through fees or community initiatives such as Jewish or Muslim burial grounds. Municipal cemeteries in the Netherlands are financed by the municipality and through grave fees, whereas commercial and religious cemeteries have mixed-models of financing through grave fees, (religious) organizations, and government support. In Sweden, grave space and maintenance of cemeteries is covered by a burial fee, paid for by an individualised tax equal to 2.5% of a person’s income, for the first 25 years use of the grave (this is a cost that everyone with an income pays, regardless of grave ownership). If the grave owner wants to extend the contract beyond 25 years, he/she pays a fee of about 100 euro for an additional 15 years. In Norway, the financing of cemeteries is part of the municipal budget. However, as the organisation responsible for burial and cremation, the Norwegian Church requests funds from the municipality annually to cover overheads and develop new burial space. In Norway the municipality also covers the cost for grave space for the first 20 years through general municipal taxes. If grave owners want to extend the contract, there is a cost of 35 euros annually. In both Scandinavian countries the cost for extending contracts vary between municipalities. In Table 2 we give an overview of the average grave costs in all case towns, however these numbers are not representative on national levels as burial costs vary across towns within countries. Costs for cremation also vary between and within countries. As an example, in Norway it is decided at a municipal level *if* and how much they want to charge for cremation, whereas in Sweden cremation is also covered by the burial fee. In the Netherlands, each crematorium has its own prices for cremation. Similarly, Luxembourgish crematoria decide fees themselves. In Scotland, cremation and burials are privately funded except when the deceased or their family have no financial means in which case the municipality is required by law to provide a public health funeral . This is typically a cremation, which is cheaper than a burial. In Ireland we see the same, both cremations and burials are privately funded, unless a family does not have the means, in which case they can apply for assistance from the state.

### Provision for Minority Communities

Across the case towns we find various examples of cemeteries and crematoria gardens that provide specific sections for religious and minority communities. However, the extent to which these provisions are planned and regulated varies. In Scotland and Ireland there is no formal requirement for designated minority religious sections to be provided within the municipal or other cemeteries and crematoria gardens; instead, these are provided on an *ad hoc* basis in response to actual or perceived local demand, and provision for minorities is widespread. In Dundee, Muslim sections within municipal cemeteries were developed through Muslim communities opening dialogue with the local authorities to request dedicated spaces. A member of the Dundee Muslim Cemetery Trust explained:

The City Council has always provided burial facilities for ethnic minorities. Different religious groups, whether it’s the Jewish quarter or the Muslims. […] In those days there were very few Muslims working in the city, but they got a hold of someone working in the factories and they said look we’ve got a Muslim person who’s passed away, what shall we do? And that’s where it came from, the area for Muslim burials.

In the Netherlands and Norway, the law states that everybody – regardless of (religious) background – has the right to be disposed after death with respect to religious needs. Furthermore, in Sweden the organization responsible for burial services (the Church) must, according to burial laws, offer special graves to others than the Christian belief. However, in practice it is up to the communities to contact the cemetery management and address their need for special grave space.

In Luxembourg-city, the mapping of cemeteries revealed that very few sections within the municipal cemeteries are based on religious or ethnic difference. Only one cemetery had separate Jewish and Muslim sections and could also be used by members of these communities residing in other parts of the country. When asked about the potential need for further minority sections, a member of staff from the local authority in charge of the city’s cemeteries explained:

There are other communities. Buddhists, but they are integrated. Then there are the Protestants, they are buried everywhere, they don’t have their own section. I think for the Muslim community, they have another approach [to burial]. […] They wanted an area, and that’s not a problem. But the other communities are integrated.

The quote shows that there is no objection to arranging separate cemetery sections. However, in Luxembourg-city (as well as in several other towns, such as Drammen, Eskilstuna and Umeå), the existence of sections for minority communities seems based on whether religious or ethnic minority communities actively demand a separate section, it is not an offer that is initiated top down, by the cemetery managers.

### Various Burial Types and Handling of Ashes

Within the study, cemeteries and crematoria gardens have different arrangements for the burial and dispersal of ashes and remains. The different sections and the types of burial permitted vary greatly not just between the countries or municipalities under study, but also across the towns. The burial of coffins or urns is most common across our cases and can be in individual or family plots. These are generally, although not always, marked with a headstone, slab, or small plaque memorial. The scattering of ashes and collective memorials are also common, reflecting high cremation ratios (see Table 2).

The presence of columbaria varies across the case studies. In the Netherlands columbaria appear more established. In the Dutch case study towns, at least half of all cemeteries have columbaria, and columbaria can be found in almost all crematoria gardens. In Luxembourg-city, only one cemetery (Merl) has a columbarium. The second columbarium to be built in Ireland was opened in Cork about 20 years ago. Various reasons may explain the historic lack of columbaria: in Ireland cremation rates are very low and only a recent option for the dispersal of bodies, whereas in Scotland they are simply not a typical cemetery architectural form despite high cremation rates, and so columbaria are not found in Dundee. In Norway, the Norwegian burial law only opened up the possibility to offer interment in columbaria on the first of January 2021. In Sweden, columbaria are rare, and there are no columbaria in the case towns under study. We now leave columbaria and turn our interest to practices around ash scattering.

In Luxembourg, it is possible to spread ashes at the national crematorium garden (located in Luxembourg-city) or sections for ash-scattering within cemeteries. This crematorium garden includes two large green lawns where ashes are scattered around trees. This is done anonymously: the names of the deceased are nowhere to be read. Leaving offerings other than flowers in these areas is not permitted and any other objects are removed by the crematorium management when placed there. In Cork, there is no provision for crematoria gardens or ash-scattering sections which reflects the low cremation rate in Ireland. However, ashes can be scattered or interred in burial plots within cemeteries. In Dundee, the various sections within the crematorium garden relate to the size and style of memorial, and again it is possible to scatter or bury cremated remains in grave plots at municipal cemeteries, it is also legally permitted to remove cremated remains from the crematoria, allowing private arrangements for storage or dispersal. In Sweden, there are spaces within cemeteries for collective unmarked urn burial of cremated remains with a collective memorial stone or sculpture nearby. In both Norway and Sweden collective memorials that include the names of the deceased posted on a wall or sculpture have been erected to allow personal identification of the deceased in situ, even if the exact place of interment remains unmarked. Interestingly, whilst preference for naming the deceased in situ is increasing, the need to identify the exact position of the remains is decreasing, in favour of eschewing long term responsibility for a plot. A cemetery worker in Umeå described this shift away from individual plots, but also noted continued visitation and mourning rituals, as well as social interactions between mourners, at the communal memorial:

The decrease in anonymous memorials is in favour of named memorials. There is also a reduction in individual urn graves in favour of collective urn memorials. […] Those [people] I meet would like to be present and bury the urns themselves. They are now allowed to do so in anonymous memorial burials, and they have thought of their descendants, their children or so, that they should not have an obligation to visit the grave or take care of it. They can come to the named memorial if they want and light a candle or leave a flower. There is no responsibility for maintenance. Instead, when they feel like it, they can come here and visit. … Then we see that it is a social thing to have relatives in a named memorial, because we observe that many older people go there often, and they get to know others who also go often. It is not the same with an individual urn grave. There, the graves are more spread-out at the cemetery, so there’s not the same chance you’ll meet someone you may have come to know a little bit. And then maybe they feel a need for talking about their relatives who have passed away, and exchange similar stories and so. So, we observe that they often talk to each other when visiting the named memorial.

Rules and practices around the handling of ashes, other than scattering, is a topic where we find clear differences across countries. In the Netherlands, Ireland, and Scotland ashes can be buried in cemeteries or scattered in crematoria gardens and memorial gardens. People can also take the ashes home, or spread them in nature such as in the sea, a river, or even in their own garden. It is even possible to split the ashes, hence dividing them between different spaces, people or objects (such as jewellery). By contrast, in Luxembourg, Sweden, and Norway, the personal disposition of cremated remains is prohibited, leaving people with no option than to scatter or bury them in cemeteries or crematoria gardens. However, the governments of Sweden and Norway both have formal processes to apply for ashes to be spread in nature (mainly in the sea, but also over land in remote areas). So far, only a small number of people have taken up this option in these countries. Across our cases, the repatriation of ashes to countries of origin or heritage is also relatively common for some migrant or minority communities, for example for Hindus and Sikhs who require cremation and dispersal over moving water, preferably the Ganges, and for East Europeans who were temporarily resident in the case study towns at the time of death (for the UK see Maddrell, Beebeejaun, McClymont, McNally, et al., 2018; for Norway see Hadders, 2021).

### The Re-Use of Graves

The possibility of re-using graves is the norm in some countries and some cultural practices, but highly contentious, taboo even, in others. In Ireland and Scotland, the re-use of grave plots is highly unusual, although not legally forbidden. In Cork, when asked about the length of grave rights and re-use of graves, interviewees, echoing Mary cited at the beginning of this paper, responded with genuine confusion, and it was necessary to explain that this cemetery management practice took place in other countries. In Dundee, one interviewee from the Council explained that an attempt to introduce grave re-use had been unpopular:

In the early 1970s they used to do grave re-use. They’ve talked about it [for the future] but I don’t think they’ll come round to doing that again. Ground will have to become really scarce before they’d start doing that.

However, in both cases, multiple burials of family members in single graves are common. By contrast, in Luxembourg, the Netherlands, Norway and Sweden, it is the cultural norm and common practice to re-use grave plots. However, there are local differences in how this is practiced across the case studies, including within specific cemeteries and cemetery sections. In Luxembourg-city, graves are emptied at the end of the rental period if the tenure is not renewed. The stone is typically removed (with only about 10 percent are retained as cenotaphs for heritage reasons), and headstones cannot be re-used or sold. The remains of the deceased are taken from the grave and transported to the communal ossuary at Fetschenhof Cemetery, allowing new burials to take place in the cemetery. In Drammen (Norway) and Eskilstuna (Sweden), the headstone is removed if the lease is not extended after the original 20 respectively 25 years lease Subsequently, if grave space is needed, the grave plot can be opened and any remains left from previous burials are put back into the ground before a new coffin is buried on top. In Umeå (northern Sweden), there are no issues with a lack of space, hence they do not currently practice re-use in the same manner. Instead, if soil conditions allow, three coffins can be placed on top of each other (as is also common in UK family graves). When a grave is re-used, the old headstone is removed and destroyed (or can be sold and re-used). Once full, new grave space is provided elsewhere in the municipality, which in practice means that grave space in Umeå is eternal even if the latest headstone has also been removed after the period of tenure.

This majority cultural norm and practice of grave re-use is problematic for some religious communities or cultures e.g. Muslims for whom perpetuity of the grave is deemed essential for the peace of the dead (Maddrell, McNally, et al., 2021). As a result, some of the case study towns do not practice the re-use of graves in specific designated religious burial areas. For example, it is stipulated in Dutch funeral law that Jewish cemeteries in the Netherlands can offer perpetual graves in contrast to majority regulation; this national regulation has not yet been extended to other minority groups, e.g., Muslim, communities. In other contexts, such as Umeå, perpetuity is not officially granted, but unofficial *ad hoc* practice accommodates minority needs given that current available grave space exceeds demand. A cemetery worker explained:

We never re-use Muslim graves. [This is] partly because we have only dug 1.5 meters deep, which means we cannot bury another coffin [there is no space on top]. And I do not think they would appreciate it.

Hence, out of respect for the Muslim beliefs and for practical reasons, Muslim graves are never re-used in the sole Muslim section in Umeå, but headstones are removed after the period of tenure.

## Discussion

This international comparative study shows that interment in cemeteries and crematoria gardens, as well as wider practices around death in Northwest Europe, differ by country and town. Despite general commonalities across our countries in terms of similar role of cemeteries, religious-cultural heritage, and post-secular contexts, this study evidences variety, both between and within these Northwest European countries. Differences include among others models for the management of cemeteries and crematoria; the possibility of perpetual grave rights; dedicated and even community-managed minorities cemetery sections; differences in the regulation of cremated remains; different practices around the re-use of grave space and cost of grave space. An explanation for these differences between countries can be found in part in the countries’ national laws and policies around burial and handling with ashes. Other factors at play are cemetery and crematorium management, cultural norms, as well as the needs and wishes of the cemetery and crematoria garden user communities. In this discussion we focus on some of the main differences and show how they impact aspects of accessibility, inclusion and equality as three characteristics which are central to the governance of public services, including cemeteries and crematoria in democratic welfare-state societies (Maddrell et al., 2021).

In the analysis of management structures we applied the three-tier management model proposed by Walter (2005), which has been insightful when looking at cemeteries and crematoria gardens from a national, top-down level. However, upon closer inspection of local cemetery and crematorium management, more complex and sometimes ad hoc social, institutional and infrastructural relations are foregrounded (Maddrell, McNally, et al., 2021) highlighting some of the limitations of the model and opening up possibilities for future research. For instance, in Scandinavia, although the Swedish or Norwegian Christian Churches run cemeteries, according to the National Burial Acts, they have to facilitate burial for all regardless of faith, which exemplifies aspects of multicultural societies, but raises questions as to whether this is truly inclusive symbolically and practically. Furthermore, we have seen some municipal cemeteries, such as those in the Netherlands, which might be assumed to be more secular, offering different sections for specific faith communities, such as Jews and Muslims; likewise in Scotland and Ireland where municipal cemeteries commonly include Christian consecrated ground and other faith sections. In effect, secular municipal cemeteries providing dedicated cemetery sections for different faith groups is a common practice which fosters inclusion and recognition of varied faith communities and their religious requirements. Echoing Maddrell, Beebeejaun, McClymont, Mathijssen, et al.’s (2018) and Maddrell, Beebeejaun, McClymont, Mathijssen, et al.’s (2018) study of England and Wales, this international comparative study shows that there are inequalities in provision for minority communities and that is typically dependent on five key factors: i) national regulation, ii) the existence of minority communities in a locality, iii) on the wishes/needs and the power of specific minority communities, iv) on the political willingness to address those needs, and v) the availability of cemetery space. National regulation is crucial, but to the extent that any arrangements for provision are *ad hoc*, e.g. relying on personal contacts, such a system creates unpredictable and unequal provisions between towns, even within the same country or town, as it depends on contingencies such as local goodwill and initiative rather than automatic citizens’ rights. There is therefore a need for clearer policy and directives at national levels on how to ensure appropriate inclusive provision for minorities at cemeteries and crematoria gardens in Northern Europe. We argue that allowing for individual practices and needs strengthens the inclusive character and accessibility of cemeteries and crematoria gardens, increasing their democratic function.

Differential regulation and practices for the disposition of cremated remains is another issue which offers insights. Some countries such as the Netherlands, Ireland, and Scotland apply a liberal approach in which ashes can be scattered in memorial gardens, in nature, or even be taken home or split in parts, to allow family members to keep the remains with them. Other countries, such as Luxembourg, Sweden, and Norway, have a much more regulated approach of ashes having to be buried or scattered at specific localities. The freedom to scatter cremated remains without regulation in the UK has raised some localized social and environment issues, particularly in favoured beauty and leisure destinations such as national parks and mountain tops (Maddrell, 2010). Yet in the context of mobile, transnational societies in which people live their lives in multiple locales, having the opportunity to transport cremated remains, and/or being allowed to scatter ashes at places of personal importance, ensures accessibility, mobility, inclusion and equality.

A final issue is that of cost in relation to inequality. Direct international comparison of cemetery and crematoria garden costs is problematic due to different average salaries, living costs and taxation regimes. Nonetheless, the implications of differences between burial and cremation costs, and personal payment versus funding through taxation, highlight actual and potential inequalities. This study clearly shows that costs and manner of payment for funerary services varies significantly internationally, ranging from high individual fees and occasional state provision for the poor, to automatic state provision funded through taxation. In those countries where burial is expensive, this has an unequal impact on certain individuals or communities who feel obliged to bury their dead, for example for religious reasons, and can cause “infrastructural harm”, i.e. harm through inadequate infrastructure, to minorities (Maddrell, McNally, et al., 2021). Costs for cremation also varies, in Scotland and Ireland cremation is approximately half the price of burial, but in Norway where burial is funded by the municipality and cremation less common outside cities, choosing cremation can incur costs, which has implications for those whose religion requires cremation, notably Hindus and Sikhs. Questions of inequality also include the merits of a one time high individual cost, e.g. €3500–4600 for a burial and funeral in Scotland, versus what is effectively payment by instalment via taxes, as is the system in Sweden where citizens pay flat rate 2.5% tax during a full working life, typically equivalent to approximately €3,900.

The complex findings of this comparative international study of cemetery and crematoria garden regulation and management, and the implications for equality-inequality, raise issues and provide insights for both scholars and practitioners. We hope the examples of good practice of minority inclusion presented in this paper highlight the need for and inspire policy and practice towards more inclusive cemetery and crematoria services across Northern Europe.

## Conclusion

In this comparative study focusing on Northern Europe we have explored similarities and differences in cemetery and crematoria management and practices around burial and ash scattering, as well as minority provision. Through a number of examples, we showed that there is variation in practices not only between countries but also between localities *within* countries, which can create unequal provision for citizens of the same country. Although we are not suggesting every cemetery and crematoria garden should create provision for every minority need regardless of local demand, but we do call for i) increased liaison between cemeteries and crematoria management and local minority communities; and ii) clearer directions on inclusive cemeteries and crematoria design and planning at national levels. Examples of such directions can be national rules with regards to facilities for washing of dead bodies, which is common practice in some communities, or allowing mourners to be present at the crematorium during cremation, both examples that differ across towns within countries, given our observations that what may seem common practice in some contexts is prohibited or contentious in other contexts. The main divergences across the nations and towns under study are the handling of ashes, the re-use of grave space and costs for burial and cremation. Below we pinpoint three key aspects of funeral practices that needs further attention in research and practice and that have an impact on accessibility, inclusion and equality.

First, the liberal approach to handling of ashes as applied in Netherlands, Ireland and Scotland adds to increased accessibility, inclusion and equality in several aspects, not only for Hindu and Sikh communities who require cremation, but also to the majority population. This becomes particularly relevant in the Scandinavian cases where cremation statistics are high, but national rules around the movement of ashes are conservative. Hence, we highlight this as a point for further discussion, not only in the countries part of this study, but more broadly in the Northern European context.

Second, the re-use of grave plots is common practice in all the studied nations except for Ireland and Scotland, which has particular impact on religious communities whose faith requires a perpetual grave. Further, how grave re-use is practiced varies across the case towns even within countries. In times of urban densification and, in some places, lack of grave space, *if* and *how* to re-use graves, are topics which urgently merit further national, local and scholarly discussion, in consultation with varied faith and community groups.

Third, as evidenced above, costs for grave plots as well as cremation varies across and within nations. This has an unequal impact on different religious communities and is particularly detrimental to low paid members of minority communities whose faith requires a specific mode of disposition which is more expensive in their country/locality. We argue that tax-funded universal funeral provision is the most inclusive financial system, compared with one time high individual cost.

To conclude, all aspects of cemetery and crematoria provision which have an unequal impact on accessibility, inclusion and equality are of particular importance to consider, given the sensitive and emotional nature of death and bereavement, and the symbolic cultural and/or religious significance of cemeteries and crematoria gardens.

## Notes

1 The legal frameworks in the countries under study are: Ireland: Regulation of Burial Grounds 1888. Luxembourg: Loi portant réglementation de l’inhumation et de l’incinération des dépouille mortelles (1972). Netherlands: Wet op Lijkbezorging (1991). Norway: Lov om gravplasser, kremasjon og gravferd (gravferdsloven) (1997). Scotland: Burial and Cremation (Scotland) Act 2016. Sweden: Begravningslagen (1990).

2 Number of stakeholder interviews: Luxembourg (N = 6), Maastricht (N = 8), Leeuwarden (N = 4), Cork (N = 7), Dundee (N = 6), Umea (N = 5), Eskilstuna (N = 4), Drammen (N = 5).
